# A Novel Data-Driven Multi-Agent Reinforcement Learning Approach for Voltage Control Under Weak Grid Support

**DOI:** 10.3390/s25237399

**Published:** 2025-12-04

**Authors:** Jiaxin Wu, Ziqi Wang, Ji Han, Qionglin Li, Ran Sun, Chenhao Li, Yuehan Cheng, Bokai Zhou, Jiaming Guo, Bocheng Long

**Affiliations:** 1 State Grid Henan Electric Power Company, Zhengzhou 450052, China; 2 College of New Energy, Harbin Institute of Technology at Weihai, Weihai 264200, China; 3 Electric Power Research Institute of State Grid Henan Electric Power Company, Zhengzhou 450052, China

**Keywords:** distribution network, distributed photovoltaics, distributed control, multi-agent deep reinforcement learning, barrier function

## Abstract

To address active voltage control in photovoltaic (PV)-integrated distribution networks characterized by weak voltage support conditions, this paper proposes a multi-agent deep reinforcement learning (MADRL)-based coordinated control method for PV clusters. First, the voltage control problem is formulated as a decentralized partially observable Markov decision process (Dec-POMDP), and a centralized training with decentralized execution (CTDE) framework is adopted, enabling each inverter to make independent decisions based solely on local measurements during the execution phase. To balance voltage compliance with energy efficiency, two barrier functions are designed to reshape the reward function, introducing an adaptive penalization mechanism: a steeper gradient in violation region to accelerate voltage recovery to the nominal range, and a gentler gradient in the safe region to minimize excessive reactive regulation and power losses. Furthermore, six representative MADRL algorithms—COMA, IDDPG, MADDPG, MAPPO, SQDDPG, and MATD3—are employed to solve the active voltage control problem of the distribution network. Case studies based on a modified IEEE 33-bus system demonstrate that the proposed framework ensures voltage compliance while effectively reducing network losses. The MADDPG algorithm achieves a Controllability Ratio (CR) of 91.9% while maintaining power loss at approximately 0.0695 p.u., demonstrating superior convergence and robustness. Comparisons with optimal power flow (OPF) and droop control methods confirm that the proposed approach significantly improves voltage stability and energy efficiency under model-free and communication-constrained weak grid conditions.

## 1. Introduction

With the continuous growth of energy demand and the advancement of the “dual carbon” goals, distributed energy resources (DERs) represented by photovoltaics (PV) and wind power have been widely integrated into distribution networks due to their cleanliness, cost-effectiveness, and operational flexibility. As a vital supplement to the main grid, the distribution network can leverage the complementary characteristics of multiple distributed energy sources to improve overall energy utilization efficiency [1].

However, large-scale PV integration alters power flow patterns in distribution systems—from unidirectional to bidirectional flow—and transforms the network topology from a radial to a multi-source coupled structure, thereby significantly increasing the complexity of system operation and control. At the same time, the high proportion of power electronic interfaces in such systems weakens their disturbance resilience, making voltage fluctuations and power quality issues increasingly prominent.

Under weak voltage support conditions, traditional voltage regulation devices struggle to achieve fast and accurate voltage control. In contrast, PV systems, owing to their flexible active/reactive power regulation capabilities, have become a promising means to improve voltage stability [2]. Thus, achieving coordinated control and efficient utilization of PV resources in weakly supported grids to ensure voltage safety and operational stability has become a key issue in both power system research and practical engineering applications.

To enable active voltage control and further enhance system stability, researchers have extensively investigated voltage regulation in smart grids. Reference [3] provided a comprehensive review of voltage optimization techniques in active distribution networks in 2024, highlighting the shift from analytical methods to computational intelligence for handling high renewable penetration. In AC distribution systems, traditional voltage regulation equipment—such as on-load tap changers (OLTCs) and shunt capacitor banks—is typically installed at substations. For instance, Reference [4] demonstrated that by optimally coordinating these devices, nodal voltages could be maintained within the statutory range of 0.95–1.05 p.u. even under heavy load conditions. However, since these devices are typically installed at substations, it is difficult to effectively regulate voltages at distant nodes. Addressing this limitation, Reference [5] proposed a decentralized control scheme robust to parameter uncertainties, ensuring system stability even with sensitivity matrix variations of up to ±48% on the IEEE 123-node feeder. With the increasing deployment of PV and other DERs, utilities worldwide are now exploring the reactive and active power control capabilities of inverter-based resources to provide new avenues for voltage regulation. Reference [6] verified through simulations that such local reactive power control schemes could achieve nearly 80% of the loss reduction potential of centralized optimization while reducing voltage volatility from 0.085 p.u. to 0.035 p.u.

Against this background, droop control strategies have been widely adopted for voltage and power coordination among distributed sources. These strategies determine inverter control commands based on functional relationships between output power and reference voltage, thereby achieving proportional sharing of active and reactive power. As a result, droop control exhibits good practicality in maintaining system voltage and frequency stability [7].

To address the issue of uneven reactive power sharing in traditional droop control, [8] proposed an improved droop control strategy based on common load voltage feedback, and experimental results showed that this method raised the Point of Common Coupling (PCC) voltage from 302 V to the nominal 311 V, effectively eliminating steady-state deviations. However, droop control relies solely on local measurements, with control parameters manually tuned and without leveraging global system information. Consequently, its performance is limited, often failing to achieve global optimality in dynamic environments. Reference [9] highlighted that traditional settings could lead to high errors, and proposed an adaptive local VVC that reduced the Mean Steady State Error (MSSE) from 4.3% to 0.3% compared to conventional methods.

To improve control precision, centralized optimization-based control methods utilize global system information to perform unified reactive power optimization, minimizing power losses while satisfying voltage constraints [10]. For instance, Reference [10] proposed a multi-timescale coordinated control method that reduced the expected energy loss to 54.9 kWh, significantly lower than the 110.3 kWh of the uncontrolled case. Reference [11] jointly optimized inverter reactive power regulation and active power curtailment to improve voltage profiles and loss performance in unbalanced low-voltage networks, successfully reducing the maximum voltage unbalance factor (VUF) to within 1%. Reference [12] employed stochastic optimization to perform centralized reactive power management considering renewable generation uncertainty, achieving algorithm convergence within 10–20 iterations with a computation time of approximately 1.2 s. Reference [13] further proposed a two-stage robust optimization model to handle uncertainty disturbances in active smart grids, ensuring system voltage stability (0.95–1.05 p.u.) even under 20% photovoltaic output uncertainty. Reference [14] proposed a three-layer reactive power hierarchical control strategy for wind-PV hybrid systems, which reduced the average system network loss by 8.14% and stabilized the collection bus voltage around 1.0 p.u. Reference [15] proposed a collaborative control method based on Analytical Target Cascading (ATC) for coupled systems, which reduced the maximum voltage deviation of terminal units to 0.0847 p.u. and achieved convergence within 8 iterations. However, centralized optimization faces significant challenges in large-scale applications due to its high computational complexity and limited solving efficiency, although Reference [16] achieved an online calculation time of approximately 3 ms through a distributed algorithm. In weakly supported grids, frequent fluctuations in DER outputs and loads cause rapid voltage variations. Since centralized control depends on global data acquisition and unified optimization, it often cannot update control commands in real time—thus failing to promptly suppress voltage deviations or maintain stability [16,17].

To overcome the limitations of centralized architectures, researchers have developed distributed optimization control frameworks. Reference [18] demonstrated that distributed control could increase distributed generation penetration by 72–86% compared to traditional power factor control. Reference [19] proposed a voltage control architecture combining local inverter reactive control with distributed cooperative control, completing voltage recovery within approximately 6 s in a 123-bus system. Reference [20] introduced a control method targeting active power consensus by jointly managing electric vehicle (EV) storage energy and PV curtailment for voltage control, which successfully maintained nodal voltages within the safe range of 0.95–1.05 p.u. and significantly mitigated voltage unbalance. Reference [21] designed a two-stage distributed consensus control strategy that first adjusts inverter reactive power and then modifies storage active power. Simulation results indicated that this strategy reduced the required energy storage capacity to 32.78% of that required by a single-stage storage control, while reducing network losses by approximately 58.5%.

Reference [22] decomposed the mixed-timescale voltage control problem into minute-level scheduling and real-time control, employing a distributed optimization framework for multi-level coordination. This method reduced the average voltage deviation by 13.25% compared to traditional centralized control and achieved a voltage violation probability as low as 0.13%. Reference [23] further proposed a multi-timescale coordination strategy combining day-ahead distributed optimization and real-time reactive/voltage control, achieving a millisecond-level online response speed (18.61 ms) and reducing daily network losses to 1615.77 kWh through a two-stage distributed coordination mechanism.

Although these distributed optimization approaches improve computational efficiency and scalability, in high-PV-penetration scenarios the system requires high-frequency communication to continuously coordinate inverter reactive power for voltage balance—posing stringent demands on communication bandwidth and latency. Moreover, in weakly supported grids, local disturbances may induce voltage amplification effects, making distributed control more sensitive to communication interruptions and topology changes. These issues constrain its applicability in large-scale, real-time voltage control [24].

Additionally, both centralized and distributed optimization methods heavily rely on accurate physical models of the distribution network. In reality, distribution systems often suffer from limited observability, parameter uncertainty, and incomplete topology information, especially in low-voltage, weak-support grids. Parameters such as line impedance, load distribution, and inverter connection details are often difficult to obtain accurately, leading to discrepancies between models and actual operation. These modeling inaccuracies weaken optimization feasibility and control performance. Furthermore, both approaches depend on intensive real-time communication and data exchange, which are difficult to guarantee in low-bandwidth, low-sampling-rate environments—further limiting their practical deployment.

However, optimization-based methods often rely on accurate physical models and parameters, which are difficult to obtain in practical distribution networks due to limited observability and time-varying topology. Furthermore, the heavy computational burden of iterative calculations limits their application in real-time control scenarios. To address these challenges, data-driven approaches have gained attention for their model-free characteristics and fast inference capabilities. For instance, Reference [25] proposed a real-time cooperative voltage regulation strategy using Approximate Dynamic Programming (ADP), which achieved an optimization accuracy of 99.90% and reduced the voltage offset by 54.6% compared to single-device regulation. In the field of reinforcement learning, Reference [26] proposed a Safe Deep Reinforcement Learning (SDRL) framework considering demand response in 2025, which successfully eliminated voltage violations (0 violations) in the IEEE 33-bus system compared to the standard Soft Actor-Critic (SAC) algorithm.

In recent years, multi-agent deep reinforcement learning (MADRL) has emerged as a rapidly developing artificial intelligence technique and a research frontier in smart grids and energy internet systems [27]. Unlike traditional optimization-based control, MADRL does not require an explicit grid model; instead, agents autonomously learn optimal control policies through interactions with the environment. The CTDE framework allows global information to be used during training, while agents make decisions independently based on local observations during execution. This eliminates real-time communication delays and bandwidth burdens. Once trained, the system can achieve cooperative PV control and voltage support with minimal communication overhead, demonstrating strong adaptability and robustness in high-penetration renewable and low-observability grids.

This paper proposes a MADRL-based photovoltaic cluster voltage coordination control method to enhance voltage stability and control intelligence in weakly supported smart grids under fluctuating PV outputs. The main contributions are as follows:

1. A Dec-POMDP-based PV coordination control framework is established, where MADRL enables distributed intelligent decision-making and cooperative optimization among multiple inverters. The framework achieves autonomous voltage support without relying on complete grid models or real-time communication, while protecting the operational data and privacy of individual PV units.
2. Two barrier functions are designed to guide agents’ learning during voltage violation events, ensuring a balance between voltage safety and control smoothness, thereby enhancing the feasibility and robustness of the learned policies.
3. Six representative MADRL algorithms are compared and analyzed in terms of voltage control accuracy and convergence speed.

The remainder of this paper is organized as follows: Section 2 develops the mathematical model of voltage control in distribution networks. Section 3 presents the proposed MADRL-based voltage control method, including the Dec-POMDP formulation and barrier-function-based learning strategy. Section 4 provides case studies to validate the effectiveness of the proposed approach. Section 5 concludes the paper and discusses future research directions.

## 2. Modeling of Voltage Control in Distribution Networks

### 2.1. Principle of Photovoltaic Participation in Voltage Control

**Figure 1:**
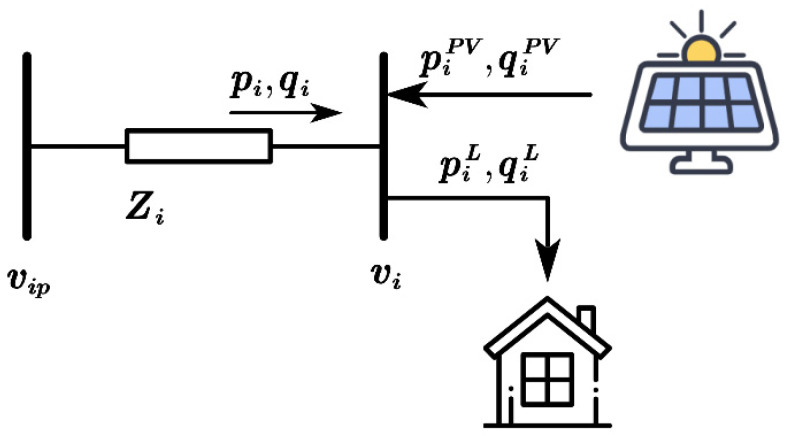
Two nodes in the distribution network.

To intuitively illustrate how photovoltaic (PV) generation affects bus voltage and how PV inverters participate in voltage regulation, consider a two-bus distribution network, as shown in Figure 1. In this network, $z_{i}=r_{i}+jx_{i}$ denotes the impedance of branch i, where $r_{i}$ and $x_{i}$ represent the resistance (in Ω) and reactance (in Ω) of the branch, respectively. $p_{i}^{L}$ and $q_{i}^{L}$ denote the active and reactive power consumption of the load (in kW and kVar), while $p_{i}^{PV}$ and $q_{i}^{PV}$ represent the active and reactive power outputs of the PV generation (in kW and kVar). $v_{ip}$ is the reference voltage at the bus expressed in per-unit (p.u.).

According to the voltage drop formula, the voltage drop across the network $\Delta v_{i}$ can be expressed as

(1) $$\Delta v_{i}=v_{ip}-v_{i}=\frac{r_{i}\left(p_{i}^{L}-p_{i}^{PV}\right)+x_{i}\left(q_{i}^{L}-q_{i}^{PV}\right)}{v_{i}}$$

From (1), it can be observed that when the bus voltage $v_{i}$ changes due to variations in load consumption or PV output, it is possible to maintain voltage stability by controlling the PV inverter to absorb or inject reactive power $q_{i}^{PV}$.

The network power loss $P_{\mathrm{loss}\;}$ can be expressed as

(2) $$P_{\mathrm{loss}\;}=\frac{{\left(p_{i}^{L}-p_{i}^{PV}\right)}^{2}+{\left(q_{i}^{L}-q_{i}^{PV}\right)}^{2}}{v_{ip}^{2}}$$

It should be noted that in (1) and (2), the only controllable variable is $q_{i}^{PV}$, which represents the reactive power generated (or absorbed) by the PV inverter. From (1), to achieve zero voltage deviation, the reactive power must satisfy:

(3) $$q_{i}^{PV}=\frac{r_{i}}{x_{i}}\left(p_{i}^{L}-p_{i}^{PV}\right)+q_{i}^{L}$$

Since $\frac{r_{i}}{x_{i}}$ is usually large in distribution feeders, during periods of peak PV generation (i.e., when $p_{i}^{PV}\gg p_{i}^{L}$), $q_{i}^{PV}$ may become significantly negative, meaning that the inverter absorbs reactive power.

From (2), to minimize power losses, $q_{i}^{PV}$ should ideally be equal to $q_{i}^{L}$; i.e., the inverter should supply reactive power. This, however, may conflict with the voltage regulation objective expressed in (3). Therefore, even for a simple two-bus network, it is difficult to simultaneously maintain voltage within safe limits and minimize power losses.

### 2.2. Smart Distribution Network Modeling

In this study, the distribution network is modeled as a multi-agent network G=(V,E), where V={0,1,…,N} and E={1,2,…,N} denote the sets of nodes and branches, respectively. Node 0 is considered as the connection point to the main grid.

For each node i∈V, let $v_{i}$ and $\theta_{i}$ denote the magnitude and phase angle of the complex voltage, respectively, and let $s_{i}=p_{i}+j\;q_{i}$ denote the complex power injection. The active and reactive power injections at node i can then be defined as

(4) $$\begin{array}{l} p_{i}^{PV}-p_{i}^{L}=v_{i}^{2}\sum_{j\in V_{i}}g_{ij}-v_{i}\sum_{j\in V_{i}}v_{j}\left(g_{ij}\cos\theta_{ij}+b_{ij}\sin\theta_{ij}\right),\forall i\in V\backslash \{0\}\\ q_{i}^{PV}-q_{i}^{L}=-v_{i}^{2}\sum_{j\in V_{i}}g_{ij}+v_{i}\sum_{j\in V_{i}}v_{j}\left(g_{ij}\cos\theta_{ij}+b_{ij}\sin\theta_{ij}\right),\forall i\in V\backslash \{0\} \end{array}$$

where $V_{i}:=\{j|(i,j)\in E\}$ denotes the set of nodes connected to node i. $g_{ij}$ and $b_{ij}$ are the conductance and susceptance (in Siemens) between nodes i and j. $\theta_{ij}=\theta_{i}-\theta_{j}$ is the phase angle difference (in radians) between the two nodes. $p_{i}^{PV}$ and $q_{i}^{PV}$ represent the active and reactive power outputs (kW/kVar) of the PV unit at node i (if no PV exists at node i, these terms are zero). $p_{i}^{L}$ and $q_{i}^{L}$ represent the active and reactive power demands of the load at node i (zero if no load is connected). Equation (4) describes the steady-state power flow equations, which are used to analyze system dynamics and address the active voltage control problem.

For safe and stable operation of power systems, bus voltages are typically permitted to vary within ±5% of their nominal values. If the rated voltage is $v_{0}=1.0(p.u.)$, the permissible voltage range can be expressed as

(5) $$0.95\;(p.u.)\;\leq v_{i}\leq 1.05\;(p.u.),\;\forall i\in V\backslash \{0\}$$

Combining (1) and (5), it can be inferred that when the load demand is heavy at night, the bus voltage $v_{i}$ may drop below 0.95 (p.u.). Conversely, during periods of strong solar irradiance, a high proportion of ${p_{i}}^{PV}$ may cause reverse power flow, increasing $v_{i}$ above 1.05 (p.u.).

The core characteristic of a Smart Grid lies in the deep integration of advanced sensing, communication, and automatic control technologies to achieve reliable, secure, economic, and efficient grid operation. For the weak voltage support smart grids studied in this paper, the increasing penetration of photovoltaics introduces strong uncertainty and high complexity, making traditional physical model-based control methods difficult to meet the Smart Grid’s requirements for real-time performance and adaptability. The MADRL-based voltage coordinated control method proposed in this paper is a concrete implementation of the “data-driven” and “distributed autonomy” concepts of the Smart Grid. By leveraging real-time operational data acquired from measurement devices deployed in the Smart Grid, this method endows PV inverters with intelligent decision-making capabilities, enabling them to perceive the environment and coordinate autonomously like agents. This transforms the traditional passive distribution network into an active system with self-perception and self-decision capabilities. This closed-loop control from data to decision not only enhances the system’s resilience against source-load fluctuations but also demonstrates the unique advantages of Smart Grid technologies in solving complex non-linear control problems, laying the theoretical and application foundation for the intelligent control framework presented in the subsequent sections.

## 3. Multi-Agent Deep Reinforcement Learning-Based Voltage Control in Distribution Networks

### 3.1. Decentralized Partially Observable Markov Decision Process (Dec-POMDP)

**Figure 2:**
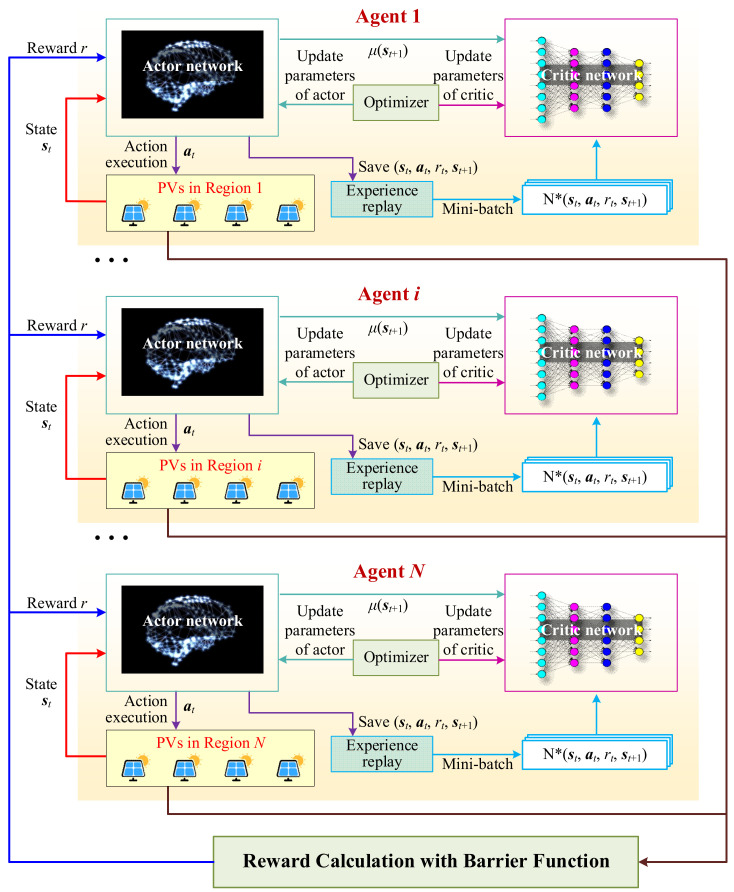
Markov Decision Process (MDP) framework diagram of proposed MADRL method.

Figure 2 provides the Markov Decision Process (MDP) framework diagram of the proposed MADRL method. Each PV region is modeled as an individual agent equipped with an actor network that receives its local state $s_{t}$, and generates a control action $a_{t}$. After executing the action in the corresponding distribution region, the resulting voltage profile is evaluated through power-flow computation, based on which a reward $r_{t}$ is calculated using the proposed barrier-function-based reward shaping mechanism. The complete transition tuple $\left(s_{t},a_{t},r_{t},s_{t+1}\right)$ is stored in an experience replay buffer for each agent. During training, mini-batches are sampled from the buffer and passed to a centralized critic network, which estimates the value function by jointly considering the global states and actions of all agents. The critic gradients are then used to update each actor network through policy-gradient backpropagation, realizing the CTDE paradigm. This process repeats across all agents (from Agent 1 to Agent *N*), forming a coordinated learning loop in which agents independently act based on local observations but learn collaboratively through the shared critic.

The goal of each agent is to maximize the cumulative discounted return $G_{t}$, as shown in Equation (6). Here, y(y∈(0,1)) is the discount factor, which discounts future rewards. The policy π followed by an agent defines the actions taken by the agent for a given state. The policy π can be either deterministic or stochastic. Equation (7) provides the expected return under policy π as the action-value function over the policy. Equation (8) gives the optimal action-value function under the optimal policy $\pi^{*}$ via the Bellman equation [28]. In DRL, the agent must follow the optimal policy $\pi^{*}$ to maximize its expected discounted reward.

(6) $$G_{t}=R_{t+1}+yR_{t+2}+y^{2}R_{t+3}+\ldots =\sum_{k=0}^{\infty }y^{k}R_{t+k+1}$$

(7) $$Q^{\pi }(s,a)=E\left[G_{t}|S_{t}=s,A_{t}=a\right]$$

(8) $$Q^{*}(s,a)=E\left[R_{t+1}+y\max_{a^{\prime }}Q^{*}\left(s^{\prime },a^{\prime }\right)\right]$$

In this paper, the power network is divided into multiple regions, each equipped with several PV generation units. Every PV unit is interfaced via an inverter capable of generating reactive power to maintain the voltage close to its reference value $v_{\mathrm{ref}\;}$. It is assumed that PV plants are owned independently of the distribution network operator, meaning that each PV owner operates its own facility autonomously.

Thus, each PV system can be regarded as an agent capable of distributed decision-making. For system safety, all agents within the same region share local observations. Since each agent can only observe partial information about the overall grid, and maintaining global voltage stability is a shared goal, the voltage control problem is appropriately modeled as a Decentralized Partially Observable Markov Decision Process (Dec-POMDP).

The MADRL problem can therefore be described as a Dec-POMDP, defined by a tuple {I,S,A,O,T,r,Ω,ρ,γ}, where I is the set of agents, and S is the set of states. $A=\times_{i\in I}A_{i}$ is the set of joint actions, where $A_{i}$ is the action set of each agent. $O=\times_{i\in I}O_{i}$ is the set of joint observations, where $O_{i}$ is the observation set of each agent. T:S×A×S→[0,1] is a state transition probability function describing the dynamics of the environment. r:S×A→ℝ is a global reward function describing the reward received by the agents after taking an action. Ω:S×A×O→[0,1] is the observation probability function, describing the probability of agents receiving a joint observation after taking an action and the state transitioning. ρ:S→[0,1] is the initial state probability distribution function. γ∈(0,1) is a discount factor. The goal of a Dec-POMDP is to find an optimal joint policy $\pi =\times_{i\in I}\pi_{i}$ that solves $\max_{\pi }E_{\pi }\left[\sum_{t=0}^{\infty }\gamma^{t}r_{t}\right]$

### 3.2. Parameter Design of MADRL for Voltage Control

(1) Agent Set

In DRL, an agent refers to an entity capable of perceiving the environment, making decisions, and executing actions. In this paper, each photovoltaic (PV) generation unit is considered an agent, with each agent located at a specific node within the power network G. The set of agents is denoted as M, and a function g:I→V is defined to map the node where each agent is located.

(2) Regional Set

This paper divides the entire power network into M control regions based on the shortest path between coupling buses and terminal buses. These regions are represented by the set

(9) $$R=\left\{R_{k}\subset V|k<M,k\in N\right\}$$

where $\cup_{R_{k}\in R}R_{k}\subseteq V$, and if $k_{1}\neq k_{2}$, then $R_{k_{1}}\cap R_{k_{2}}=\emptyset$.

A function f:V→R is defined to map the region to which a node belongs.

(3) State and Observation Sets

The state and observation sets refer to the collection of environmental variables observable by the agents. In this paper, the state and observation sets are defined as

(10) $$\left\{\begin{array}{l} S=L\times P\times Q\times V\\ L=\left\{\left(p^{L},q^{L}\right):p^{L},q^{L}\in {\left(0,\infty \right)}^{\left|V\right|}\right\}\\ P=\left\{p^{PV}:p^{PV}\in {\left(0,\infty \right)}^{\left|I\right|}\right\}\\ Q=\left\{q^{PV}:q^{PV}\in {\left(0,\infty \right)}^{\left|I\right|}\right\}\\ V=\left\{(v,\theta ):v\in {\left(0,\infty \right)}^{\left|V\right|},\theta \in {\left[-\pi ,\pi \right]}^{\left|V\right|}\right\} \end{array}\right.$$

where L is the set of active and reactive power of loads. P is the set of active power generated by PV units. Q is the set of reactive power generated by PV units. V is the set of voltages, where v represents the voltage magnitude and θ represents the voltage phase angle in radians.

A function h:P(V)→P(S) is defined to map subsets of V to their associated measurements, where P(⋅) denotes the power set. The observation set is defined as $O=\times_{i\in I}O_{i}$, where $O_{i}=(h{}^{\circ}f{}^{\circ}g)(i)$ represents the measurements within the region of agent i.

(4) Action Set

Each agent i∈I is equipped with a continuous action set $A_{i}=\left\{a_{i}:-c\leq a_{i}\leq c,c>0\right\}$. The continuous action represents the ratio of the reactive power it generates to the maximum reactive power it can produce. For example, the reactive power generated by the k-th PV inverter is

(11) $$q_{k}^{PV}=a_{k}\sqrt{{\left(s_{k}^{\max}\right)}^{2}-{\left(p_{k}^{PV}\right)}^{2}}$$

where $s_{k}^{\max}$ is the maximum apparent power (in kVA) at the k-th node, determined by the physical capacity of the PV inverter. If $a_{k}>0$, it indicates injecting reactive power into the distribution network. If $a_{k}<0$, it indicates absorbing reactive power from the distribution network. The value of c is typically chosen based on the load capacity of the distribution network to ensure operational safety. The joint action set is denoted as $A=\times_{i\in I}A_{i}$.

(5) State Transition Probability Function

Since the state includes the last action and load changes are stochastic, the state transition probability function can be naturally defined as T:S×A×S→[0,1], which follows a Markov decision process. Specifically, $T\left(s_{t+1}|s_{t},a_{t}\right)=P\left(s_{t+1}|\delta \left(s_{t},a_{t}\right)\right)$, where $a_{t}\in A$ and $s_{t},s_{t+1}\in S$. Here, $\delta \left(s_{t},a_{t}\right)\to s_{t+T}$ denotes the power flow solution, while $P\left(s_{t+1}|s_{t+1}\right)$ describes the load variation. T≪Δt is an extremely short time interval, much smaller than the time interval between two control steps (i.e., the time step). In this paper, Δt=1.

(6) Observation Probability Function

In the context of power networks, the observation probability function describes potential measurement errors from sensors. It can be defined as Ω:S×A×S→[0,1]. Specifically, $\Omega \left(o_{t+1}|s_{t+1},a_{t}\right)=s_{t+1}+N(0,\Sigma )$, where N(0,Σ) is an isotropic multivariate Gaussian distribution and Σ depends on the physical properties of the sensors.

(7) Reward Function

To ensure voltages converge rapidly to the reference voltage $v_{\mathrm{ref}}=1(p.u.)$, the reward function is defined as

(12) $$r=-\frac{1}{\left|V\right|}\sum_{i\in V}l_{v}\left(v_{i}\right)-\alpha \cdot l_{q}\left(q^{PV}\right)\;$$

where $l_{v}\left(v_{i}\right)$ is a barrier function, and $l_{q}\left(q^{PV}\right)=\frac{1}{\left|I\right|}\left|q^{PV}\right|$ represents the reactive power loss (in kVar). The control objective is to regulate voltages within a safe range around $v_{\mathrm{ref}}$ while minimizing reactive power generation, i.e., $l_{q}\left(q^{PV}\right)<\varepsilon$ with ε>0.

(8) Objective Function

The goal of DRL is to find an optimal joint policy π that maximizes the discounted cumulative return. Thus, the objective function of this problem is $\max_{\pi }E\pi \left[\sum t=0^{\infty }\gamma^{t}r_{t}\right]$, where $\pi =\times_{i\in I}\pi_{i}$ and $\pi_{i}:\bar{O}i\times Ai\to [0,1]$.

In our MADRL design, each PV inverter is instantiated as a distinct agent. Each agent receives only local voltage, load, and PV measurements and independently determines its reactive power output. The cooperative aspect arises through the CTDE framework: a centralized critic jointly evaluates all agents’ actions during training, capturing the coupling of voltage dynamics across the distribution network. Through this shared critic, agents learn policies that not only optimize local objectives but also contribute to global voltage stability. At deployment, the agents execute their learned policies in a fully decentralized manner based solely on local observations, thereby preserving scalability and robustness. This architecture explicitly embodies the multi-agent nature of the proposed method.

**Figure 3:**
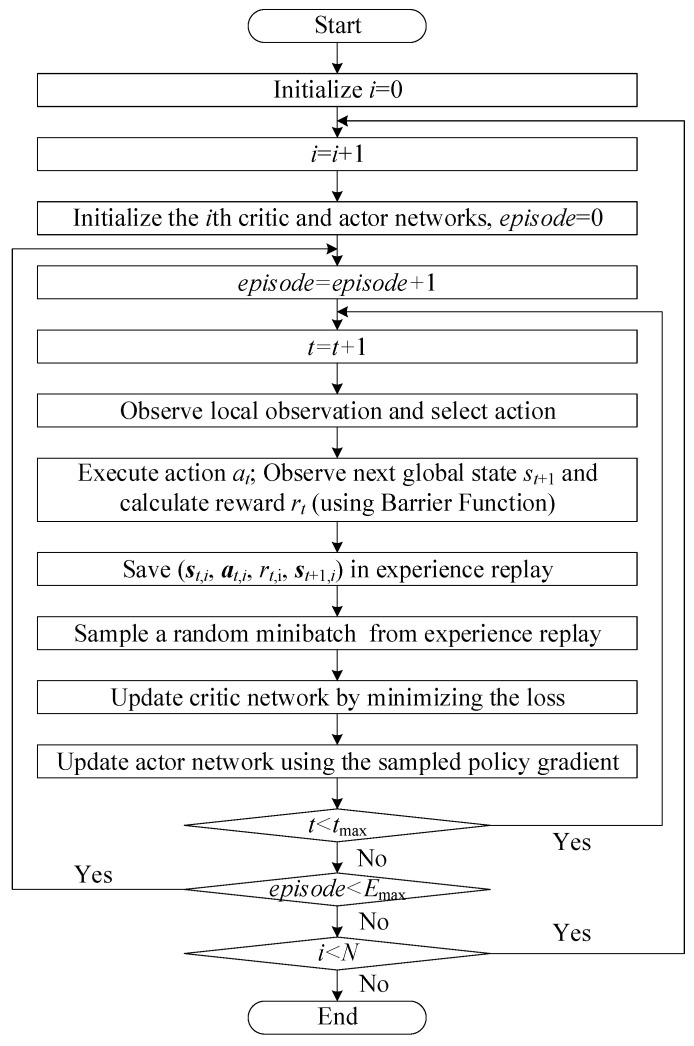
Overall control flowchart of the proposed MADRL-based coordinated voltage regulation methodology.

Figure 3 presents the overall control flowchart of the proposed MADRL-based coordinated voltage regulation methodology. The diagram summarizes the complete learning and control pipeline, starting from initialization and proceeding through agent-wise training and episodic interactions with the distribution network environment. For each agent i, the actor–critic networks are first initialized, after which the learning process iterates over episodes and time steps. At each time step, the agent observes its local state, selects an action through its actor network, and the distribution system evolves to the next global state through power-flow computation. The reward $r_{t}$ is then calculated using the proposed barrier-function-based reward mechanism, and the transition tuple $\left(s_{t},a_{t},r_{t},s_{t+1}\right)$ is stored in the replay buffer. During training, mini-batches sampled from the replay memory are used to update the critic network by minimizing the temporal-difference loss, and subsequently the actor network is optimized using the policy gradient derived from the centralized critic. This iterative updating continues until the maximum time step $t_{max}$ and maximum episode number $E_{max}$ are reached. The procedure is repeated for all agents i=1,…,N, thus completing the full CTDE-based MADRL learning cycle.

### 3.3. Barrier Function

In this paper, the reference voltage is defined as $v_{\mathrm{ref}}=1\left(p.u.\right)$, and the voltage is required to be controlled within the safe range from 0.95 (p.u.) to 1.05 (p.u.), which establishes the voltage constraints. The voltage constraint is represented by a barrier function in this study.

**Figure 4:**
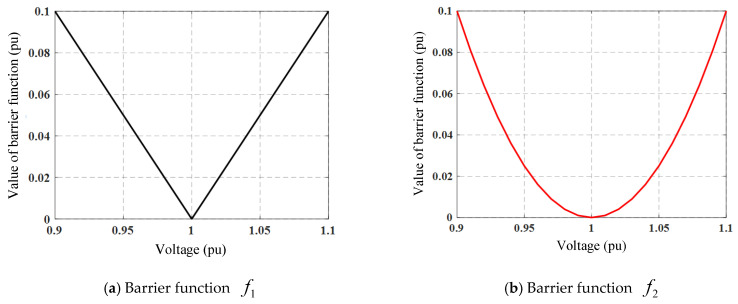
Barrier function.

As shown in Figure 4, two types of barrier function curves are illustrated, where the horizontal axis represents the voltage v expressed in p.u. and the vertical axis represents the penalty value p. The function $f_{1}$ (see Figure 4a) has been most frequently used in previous studies. However, this may lead to reactive power waste, since even when the voltage is already within the safe range of 0.95 (p.u.) to 1.05 (p.u.), the following still holds:

(13) $$\frac{\left|\Delta l_{v}\right|}{\alpha \left|\Delta l_{q}\right|}\gg 1$$

To address this issue, this paper proposes a novel quadratic-type barrier function $f_{2}$ (see Figure 4b), which provides a larger gradient when the voltage is outside the safe range, while offering a smaller gradient as the voltage approaches $v_{\mathrm{ref}}$. As $v\to v_{\mathrm{ref}}$, it satisfies

(14) $$\frac{\left|\Delta l_{v}\right|}{\alpha \left|\Delta l_{q}\right|}\to 0\;$$

The proposed barrier function $f_{2}$ can therefore provide a larger gradient outside the safe voltage range, enabling the bus voltage to quickly converge to $v_{\mathrm{ref}}$, and a smaller gradient within the safe voltage range, significantly reducing power losses as the bus voltage approaches $v_{\mathrm{ref}}$. By adopting the quadratic-type barrier function $f_{2}$, fast active voltage regulation can be achieved while maintaining excellent performance in minimizing power losses.

### 3.4. MADRL Algorithms

#### 3.4.1. Algorithm Selection and Fairness Setup

In this study, we deliberately select six representative multi-agent reinforcement learning algorithms—COMA, IDDPG, MADDPG, MAPPO, SQDDPG, and MATD3—as benchmarking baselines. These methods are widely recognized in the MARL community and collectively span the mainstream methodological categories, including value-decomposition-based methods (COMA), independent deterministic actor–critic methods (IDDPG), centralized-training–decentralized-execution (CTDE) frameworks (MADDPG and MATD3), policy-gradient-based multi-agent actor–critic methods (MAPPO), and stability-enhanced deterministic learning approaches (SQDDPG). Selecting these algorithms ensures that our evaluation covers a broad spectrum of coordination mechanisms and learning paradigms that are most relevant to multi-agent cooperative control in power systems.

Given the number and diversity of algorithms involved, it is impractical to present the complete mathematical formulations and implementation-level derivations of each algorithm within this paper. The details for each method are provided in [29,30,31,32,33,34].

To ensure strict fairness across all algorithm comparisons, all training and evaluation experiments follow identical settings. First, the objective function is kept exactly the same for all methods. Second, all algorithms operate with identical state representations, observation scopes, action bounds, reward functions, environmental parameters, and stochastic disturbances derived from real-world data. Third, hyperparameters such as learning rates, batch sizes, replay buffer sizes, target-network update rates, entropy coefficients, and training episode counts are tuned following each algorithm’s canonical guidelines while being constrained within a unified search range to avoid biased configurations. Additionally, all experiments are repeated under the same random seeds, and final results are averaged over multiple trials to eliminate randomness-induced performance variance.

The motivation for choosing these six algorithms lies in their representativeness, maturity, and complementary characteristics. Together, they enable a systematic examination of how different MARL mechanisms—credit assignment (COMA), independent learning (IDDPG), CTDE critics (MADDPG/MATD3), entropy-regularized policy optimization (MAPPO), and TD3-style twin critics with robustness (SQDDPG)—affect coordinated voltage regulation under weak voltage support conditions. This comprehensive selection not only enhances the credibility and breadth of the benchmarking results but also provides valuable insights into how different learning paradigms respond to the challenges of highly stochastic, data-driven smart grid environments.

#### 3.4.2. Algorithm Assumptions

In developing the MADRL-based voltage control algorithms, several key assumptions were made to adapt the complex distribution network control problem into a standard reinforcement learning task. First, we assume that during the centralized training phase of the CTDE framework, agents can share information without delay via high-bandwidth communication to construct the global state for Critic updates; during the decentralized execution phase, each agent is assumed to obtain all required local measurements instantaneously at its own bus, and therefore no real-time communication delay is considered. The reason for this assumption is that the proposed control policy relies only on locally measurable quantities (such as node voltage and local power injections), which can be acquired directly from field sensors without involving long-distance communication links. Second, we assume that the voltage state transition in the distribution network follows the Markov property, meaning that the voltage at the next time step depends only on the current power injections and network state, ignoring long-term historical memory effects. Finally, it is assumed that the physical topology of the distribution network remains relatively stable within a single control cycle, without considering abrupt topological changes.

#### 3.4.3. Algorithm Introduction

(1) Counterfactual Multi-Agent Policy Gradients (COMA)

Under the traditional assumption of independent decision-making, MADRL algorithms face the challenge of non-stationarity in action evaluation. The COMA algorithm addresses this issue by introducing a “counterfactual baseline” to estimate the difference in expected return corresponding to the current actor. COMA employs an Actor-Critic approach to train the actor, updating the action gradient for each agent based on temporal difference learning [29]:

(15) $$\nabla \theta^{\pi }=\frac{\partial }{\partial \theta^{\pi }}\log\pi \left(\mu |\tau_{t}^{a}\right)\left(r+\gamma \left(s_{t+1}\right)-V\left(s_{t}\right)\right)$$

where $\theta^{\pi }$ denotes actor parameters, μ denotes the actor network and $\tau_{t}^{a}$ denotes the agent observation given the environment and action.

Furthermore, COMA utilizes a counterfactual baseline for computation. This baseline ignores the actions of a single agent while keeping the actions of other agents unchanged, and uses a centralized critic to calculate the advantage function. Through this counterfactual baseline and centralized critic, COMA can effectively evaluate the advantages of agent actions in multi-agent environments, facilitating the optimization of coordination and decision-making among multiple agents in complex scenarios.

(2) Independent Deterministic Policy Gradient (IDDPG)

IDDPG assumes that multiple agents are independent of each other. Each agent treats other agents as part of the environment and learns its policy using the DDPG framework individually. For the i-th agent, its actor outputs a deterministic action $a_{i}=\mu_{\theta_{i}}\left(o_{i}\right)$, where $o_{i}$ is the local observation of the agent, and $\mu_{\theta_{i}}$ represents its actor parameters. The corresponding critic network estimates the Q-value function $Q_{\phi_{i}}\left(o_{i},a_{i}\right)$, aiming to minimize the TD (Temporal Difference) error [30]:

(16) $$\begin{array}{l} L\left(\phi_{i}\right)=E_{\left(o_{i},a_{i},r_{i},o_{i}^{\prime }\right)}\left[{\left(Q_{\phi_{i}}\left(o_{i},a_{i}\right)-y_{i}\right)}^{2}\right]\\ y_{i}=r_{i}+\gamma Q_{\phi_{i}}\left(o_{i}^{\prime },\mu_{\theta_{i}^{\prime }}\left(o_{i}^{\prime }\right)\right) \end{array}$$

where $\phi_{i}^{\prime }$ and $\theta_{i}^{\prime }$ are the parameters of the target critic and target actor networks, respectively, and γ is the discount factor.

The actor is updated via the deterministic policy gradient:

(17) $$\nabla_{\theta_{i}}J\left(\theta_{i}\right)\approx E_{o_{i}}\left[{\left.\nabla_{a_{i}}Q_{\phi_{i}}\left(o_{i},a_{i}\right)\right|}_{a_{i}=\mu_{\theta_{i}}\left(o_{i}\right)}\nabla_{\theta_{i}}\mu_{\theta_{i}}\left(o_{i}\right)\right]$$

where $\theta_{i}$ and $\phi_{i}$ are the parameters of the i-th agent’s actor and critic networks, respectively. $r_{i}$ is the reward obtained by the agent at the current step. $o_{i}^{\prime }$ is the observation of the next state. $Q_{\phi_{i}}\left(o_{i},a_{i}\right)$ is the estimated action-value function evaluating the quality of the current action. $\mu_{\theta_{i}}\left(o_{i}\right)$ represents the deterministic action output by the actor.

(3) Multi-Agent Deep Deterministic Policy Gradient (MADDPG)

The MADDPG algorithm enables decision-making in cooperative or competitive environments by jointly training the actor and critic networks of multiple agents. Through iterative updates of the parameters in the value function, agents can gradually learn coordinated actions or competitive strategies, achieving better performance. For the i-th agent, its goal is to maximize its own expected return [31]:

(18) $$J\left(\theta_{i}\right)=E_{s,a_{1},\ldots ,a_{N}\sim D}\left[Q_{i}\left(s,a_{1},\ldots ,a_{N}\right)\right]$$

where $\theta_{i}$ is the actor parameter of the i-th agent, and $Q_{i}$ is the joint action-value function estimated by the agent’s critic, depending on the environment state s and the joint actions of all agents $\left(a_{1},\ldots ,a_{N}\right)$.

The critic is trained using the following objective:

(19) $$L\left(\phi_{i}\right)=E_{s,a,r_{i},s}\left[{\left(Q_{i}\left(s,a_{1},\ldots ,a_{N}\right)-y_{i}\right)}^{2}\right]$$

where $y_{i}=r_{i}+\gamma Q_{i}^{\prime }\left(s^{\prime },a_{1}^{\prime },\ldots ,a_{N}^{\prime }\right)$, $a_{j}^{\prime }=\mu_{j}^{\prime }\left(o_{j}^{\prime }\right)$, for all j=1,…,N. $Q_{i}^{\prime }$ and $\mu_{j}^{\prime }$ are target networks used to stabilize training. $s^{\prime }$ is the next state, and $o_{j}^{\prime }$ is the next observation of agent j.

The actor’s update direction is given by the policy gradient:

(20) $$\nabla_{\theta_{i}}J\left(\theta_{i}\right)=E_{s}\left[{\left.\nabla_{\theta_{i}}\mu_{i}\left(o_{i}\right)\nabla_{a_{i}}Q_{i}\left(s,a_{1},\ldots ,a_{N}\right)\right|}_{a_{j}=\mu_{j}\left(o_{j}\right)}\right]$$

where $\phi_{i}$ is the parameter of the critic, $\mu_{i}\left(o_{i}\right)$ is the action output by the actor and $Q_{i}\left(s,a_{1},\ldots ,a_{N}\right)$ is the joint Q-function, depending on the global state s and the joint actions of all agents. D is the experience replay buffer.

(4) Multi-Agent Proximal Policy Optimization (MAPPO)

MAPPO is a MADRL algorithm that extends the PPO method to multi-agent environments, incorporating the idea of centralized training with decentralized execution. In MAPPO, each agent has its own actor, but all agents can share one or more centralized critics to capture the cooperative relationships among multiple agents in the environment. Its core objective is to maximize the expected return for each agent while maintaining the stability of actor parameter updates. The core loss function of MAPPO inherits from PPO, namely the “clipped” policy optimization objective [32]:

(21) $$L^{\mathrm{CLIP}}(\theta )=E_{t}\left[\min\left(r_{t}(\theta ){\hat{A}}_{t},\mathrm{clip}\left(r_{t}(\theta ),1-\epsilon ,1+\epsilon \right){\hat{A}}_{t}\right)\right]$$

where θ denotes the actor parameters. $r_{t}(\theta )=\frac{\pi_{\theta }\left(a_{t}|o_{t}\right)}{\pi_{\theta_{\mathrm{old}\;}}\left(a_{t}|o_{t}\right)}$ is the probability ratio of the current actor to the old actor for action $a_{t}$. $\pi_{\theta }\left(a_{t}|o_{t}\right)$ represents the probability of the agent taking action $a_{t}$ given observation $\;o_{t}$. ${\hat{A}}_{t}$ is the advantage function, measuring the relative benefit of the current action compared to the average. ϵ is a small constant (typically between 0.1 and 0.2) that limits the magnitude of actor updates. The clip(⋅) function suppresses performance degradation caused by overly rapid actor updates.

(5) Soft Q-function-based Deep Deterministic Policy Gradient (SQDDPG)

SQDDPG is a continuous action space DRL algorithm that integrates ideas from Soft Actor-Critic into the DDPG architecture. It aims to enhance the robustness and exploration capability of DDPG in complex environments, particularly suited for scenarios requiring a balance between “optimality” and “entropy”. SQDDPG introduces the maximum entropy learning framework by incorporating an entropy term into the actor optimization, encouraging more exploratory behavior from the actor. Its core objective function is to maximize the expected return of the following form [33]:

(22) $$J(\pi )=E_{\pi }\left[\sum_{t=0}^{\infty }\gamma^{t}\left(r\left(s_{t},a_{t}\right)+\alpha H\left(\pi \left(\cdot |s_{t}\right)\right)\right)\right]$$

where $H\left(\pi \left(\cdot |s_{t}\right)\right)=-\log\pi \left(a_{t}|s_{t}\right)$ represents the entropy of the current actor in state $s_{t}$, and α is the entropy coefficient, balancing exploration and reward.

The actor’s update objective is to maximize:

(23) $$J_{\pi }=E_{s_{s_{t}}\sim D}\left[E_{a_{t}\sim \pi }\left[\alpha \log\pi \left(a_{t}|s_{t}\right)-Q\left(s_{t},a_{t}\right)\right]\right]$$

(24) $$J_{Q}=E_{\left(s_{t},a_{t},r_{t},s_{t+1}\right)\sim D}\left[{\left(Q\left(s_{t},a_{t}\right)-\left(r_{t}+\gamma E_{a_{t+1}\sim \pi }\left[Q\left(s_{t+1},a_{t+1}\right)-\alpha \log\pi \left(a_{t+1}|s_{t+1}\right)\right]\right)\right)}^{2}\right]$$

where $\pi \left(a_{t}|s_{t}\right)$ is the action probability distribution output by the actor. $Q\left(s_{t},a_{t}\right)$ is the action-value function. α is the importance coefficient controlling the action entropy; a larger value encourages more exploration. H(π) is the action entropy, measuring the randomness of the actions. D is the experience replay buffer.

(6) Multi-Agent Twin Delayed Deep Deterministic Policy Gradient (MATD3)

The MATD3 algorithm is a MADRL algorithm designed to address collaborative decision-making problems. Unlike traditional Q-learning or DDPG algorithms, MATD3 combines multiple actors and twin critics to train multiple agents, thereby better optimizing the actors. In MATD3, each agent has an actor that generates actions. During training, two critic networks are used simultaneously to evaluate the quality of actions, leading to more accurate estimation of the Q-value function and improved learning efficiency for the agents. MATD3 predicts the action-value function of the next state using target networks and the current state, obtaining the TD target [34]:

(25) $$y=r+\gamma (1-d)Q^{\prime }\left(s^{\prime },\mu^{\prime }\left(s^{\prime },\theta_{\mu }^{\prime }\right);\theta_{Q^{\prime }}\right)+\gamma dV^{\prime }\left(s^{\prime };\theta_{V^{\prime }}^{\prime }\right)\;$$

where ${\theta^{\prime }}_{\mu }$, ${\theta^{\prime }}_{Q^{\prime }}$, and $\theta_{V^{\prime }}^{\prime }$ are the parameters of the functions $\mu^{\prime }$, $Q^{\prime }$, and $V^{\prime }$, respectively. $Q^{\prime }$ and $V^{\prime }$ are the target action-value function and state-value function, respectively. $\mu^{\prime }$ is the action from the target network. d is the terminal state flag.

The critic’s loss function is shown in Equation (26):

(26) $$L\left(\theta_{Q}\right)=(1/N)*{\sum \left[Q\left(s,a;\theta_{Q}\right)-y\right]}^{2}$$

where $Q\left(s,a;\theta_{Q}\right)$ is the action-value function, $\theta_{Q}$ are the policy parameters and N is the number of samples.

#### 3.4.4. Algorithm Limitations

Based on assumptions in Section 3.4.2, the proposed algorithms exhibit certain limitations in practical applications. The first is the issue of safety assurance. Although barrier functions are introduced as soft constraints to penalize voltage violations, MADRL is inherently a probabilistic exploration method. It cannot provide theoretical “hard constraint” safety guarantees during the early stages of training or when facing unseen extreme scenarios, which poses a potential risk in power systems with high safety requirements. The second limitation is the Sim-to-Real Gap. Since the algorithms are trained in a physics-based simulation environment, factors in real-world grids such as non-Gaussian measurement noise, communication packet loss, and physical response latency of inverters may lead to policy performance degradation. The final limitation concerns scalability. Although CTDE mitigates communication burdens during execution, the state space dimensionality and computational complexity of the centralized training phase grow exponentially with the significant increase in the number of network nodes and agents, potentially limiting applicability in ultra-large-scale smart grids.

## 4. Case Studies and Analysis

### 4.1. Parameter Settings

To characterize the impact of uncertainty on scheduling results, this study introduces different confidence levels α in the case study. By varying the conservatism degree of risk constraints, the operational characteristics of the system under different reliability requirements are compared. Specifically, higher confidence levels require scheduling results to remain feasible in more scenarios, making the model more conservative, while lower confidence levels allow the system to be feasible in fewer scenarios, enabling greater risk tolerance and thus improving renewable energy utilization. To comprehensively reveal this trade-off relationship, this paper sets α=0.98, 0.96, 0.94, 0.92, 0.90, and 0.88, and conducts comparative analysis of the scheduling results.

**Figure 5:**
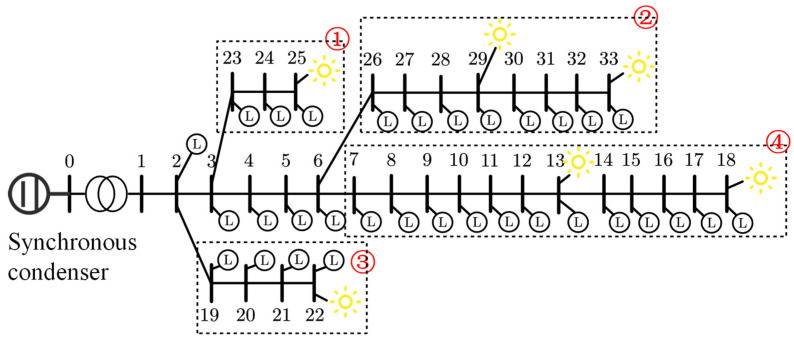
IEEE 33-bus network with photovoltaic power generation.

This case study constructs a test system based on the IEEE 33-node distribution network, with adjustments made to the original network structure to suit the research on multi-region cooperative control in smart grids. Node 0 is considered the interface point with the main grid, connected via a transformer with a rated voltage of 220 kV to a synchronous condenser with a capacity of 300 Mvar, simulating the limited reactive power support capability of the main grid under weak grid conditions. The system is divided into four control regions (indicated by the red dashed circles labeled 1–4 in Figure 5), each containing several PV access points and customer loads. Except for Node 1, all other nodes have loads; Nodes 13, 18, 22, 25, 29, and 33 are connected to photovoltaic generation units, forming a distributed source-load hybrid system. The modified topology is shown in Figure 5. Line impedances follow the standard IEEE 33-bus dataset and are expressed in Ω. Loads are represented in kW/kVar. PV generation profiles are provided in kW. The synchronous condenser is rated at 300 kVar, and the transformer secondary voltage is 220 V.

The detailed electrical parameters of the modified IEEE 33-bus network—including branch resistances/reactances, load assignments, PV inverter ratings, transformer parameters, and synchronous condenser capacity—are derived from the standard IEEE 33-bus benchmark dataset [35]. The modifications introduced in this study (i.e., PV placement, regional partitioning, and the addition of weak-grid support elements) are explicitly documented to ensure reproducibility.

The “data-driven” approach adopted in this paper extends beyond merely using real datasets for testing; it fundamentally involves constructing a high-fidelity training environment using high-granularity historical operational data to drive agents in learning robust voltage control policies. To capture the stochastic fluctuation characteristics of sources and loads in smart grids, this study utilizes two sets of high-resolution real-world datasets: load data derived from real electricity consumption records of the Portuguese national grid, comprising continuous load data for 232 users over 3 years (1096 days); and photovoltaic generation records from the Belgian grid operator Elia Group, covering 10 typical regions over the same 3-year period. Both datasets maintain a 3 min time resolution. Unlike traditional statistical distribution models (e.g., Gaussian distribution), these real-world data preserve the intermittency of PV output (such as sudden drops due to cloud cover) and the temporal correlation of user loads, providing a solid data foundation for discovering extreme voltage scenarios.

To adapt to the IEEE 33-bus topology and enhance physical realism, strict preprocessing and augmentation steps were executed before feeding data into the model. First, spatiotemporal matching was performed to randomly map the load profiles of 232 users and PV profiles of 10 regions to various nodes of the distribution network, strictly aligning them on the time axis to form corresponding active/reactive power injection matrices. Second, considering that the power factor of actual users is not constant, random perturbations within the range of [−0.05, 0.05] were introduced on top of the default power factor. This step generates time-varying reactive load data, significantly increasing the complexity and diversity of the environment state space. This forces agents to learn to cope with uncertain reactive power demands during training, thereby preventing the policy from converging to local optima specific to single operating conditions.

In the constructed IEEE 33-node distribution network, the system is divided into 4 control regions, and 6 agents are deployed to perform the MADRL task. The discount factor γ is set to 0.99 to maintain the importance of future rewards; the entropy regularization coefficient α is set to 0.1 to regulate the degree of policy exploration. To ensure the safety and controllability of the distribution system operation, the reactive power output range of the PV inverters is limited to the normalized interval of [−0.8,0.8].

Within this framework, data enhances the MADRL training process by directly constructing the state space and driving policy updates. The agent’s local observation (including node voltage, PV output, and load demand) is generated directly from the real data snapshot of the current time step. Rich data samples ensure that agents can traverse various typical and extreme states, ranging from voltage over-limit caused by “light load/high PV” to voltage under-limit caused by “heavy load/no PV”. Simultaneously, the voltage deviation fed back by power flow calculations based on real data forms the basis of the reward function. Repeated episode sampling based on 3 years of historical data ensures that the experience replay buffer stores a vast number of realistic system state transition samples. This not only stabilizes the value estimation of the Critic network but also effectively prevents policy overfitting through data diversity, thereby endowing the agents with stronger generalization capabilities when facing unknown seasonal and weather conditions.

This paper selects six typical MADRL algorithms for model training and performance comparison, including COMA, IDDPG, MADDPG, MAPPO, SQDDPG, and MATD3. During training, the initial system state is determined by random sampling at the beginning of each episode; each episode contains 240 time steps, corresponding approximately to half a day of operation in the real system. The 240 time steps correspond to 3 min intervals, matching the resolution of load and PV data. Reactive power outputs are normalized to [–1, 1], corresponding to actual kVar values determined by inverter ratings. To continuously evaluate the model’s learning progress, a test procedure is executed every 20 training episodes. During the testing phase, 10 random episodes are independently sampled from the policy, and their performance metrics are averaged to serve as a measure of the current policy’s performance under different scenarios.

For performance evaluation, key metrics (such as cumulative reward, voltage deviation, etc.) from all test episodes are statistically analyzed. The median is used as the central tendency indicator for performance assessment, and the 25–75% interquartile range is used to represent the interval confidence of the results, thereby improving the description of the algorithm’s stability and robustness under different operating conditions. The Controllability Ratio (CR) and Power Loss (PL) metrics are used to evaluate algorithm performance: CR calculates the proportion of time steps within each episode where the voltages of all buses are controlled. PL calculates the average total power loss across all buses for each time step within an episode.

The simulation environment was established on a workstation. All simulations, including the distribution network modeling, power flow calculations, and the implementation of the proposed MADRL algorithms, were performed using MATLAB R2021b (MathWorks, Natick, MA, USA).

### 4.2. Training Process

**Figure 6:**
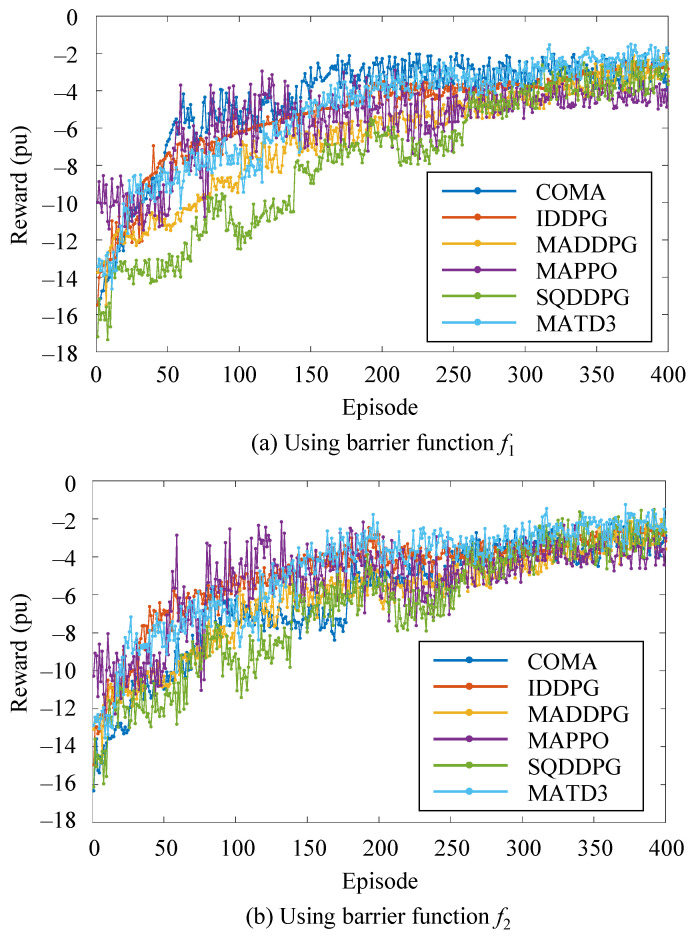
Reward value curves of six MADRL algorithms during the training process.

To better visualize the training process, the first 400 training episodes for function $f_{1}$ and function $f_{2}$ are displayed separately. The training curves for all MADRL algorithms in the 33-node network are shown in Figure 6. The reward becomes negative because the barrier-function-based design imposes penalties whenever the bus voltage deviates from the safe range, resulting in negative reward values during violation periods. According to the results shown in Figure 6, as the number of training episodes gradually increases, the reward value r for all six MADRL algorithms gradually increases. This indicates that these agents gradually accumulate experience while interacting with the PV microgrid and continuously optimize their control strategies, eventually reaching a stable state. This suggests that after a certain number of training cycles, the agents can gradually converge to a relatively stable optimal control strategy, thereby improving the control performance and stability of the PV microgrid.

### 4.3. Comparison of Different MADRL Algorithms

**Figure 7:**
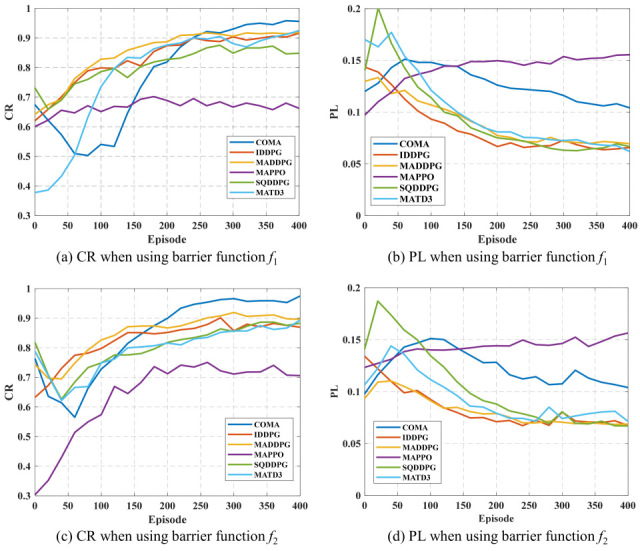
Performance of six MADRL methods on CR and PL.

To evaluate the performance of MADRL algorithms in the control task, this paper selects CR and PL as the main performance metrics. Figure 7 shows the comparison results of the six MADRL methods under these two metrics. It can be observed from the figure that IDDPG and MADDPG demonstrate superior performance in both CR and PL, achieving a good compromise between improving system controllability and reducing energy loss. In comparison, the performance of COMA, SQDDPG, and MATD3 is relatively suboptimal, but after a certain number of training episodes, both CR and PL metrics show significant improvement, indicating their potential for performance enhancement. Among them, COMA achieves the highest controllability ratio, but it is accompanied by relatively large power loss, suggesting that the trade-off strategy between control coverage and energy efficiency in this method is more aggressive. Among all the compared algorithms, MAPPO is the only method that performs poorly in both CR and PL metrics. Its performance bottleneck may stem from the conservatism in its policy update process, leading to insufficient responsiveness when dealing with the frequently changing dynamics and high uncertainty in the distribution network, thus limiting its policy’s adaptive capability.

In summary, algorithms with fast response capabilities and inter-agent modeling mechanisms (such as IDDPG and MADDPG) have advantages in weak grid environments, while algorithms like MAPPO that rely on policy clipping mechanisms may have adaptability issues in complex distributed scenarios.

### 4.4. Influence of Different Barrier Functions

**Figure 8:**
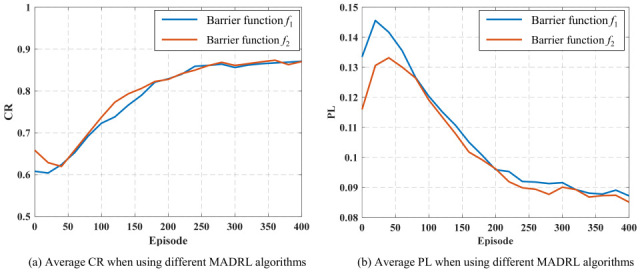
Average CR and PL of six MADRL algorithms under different obstacle functions.

To analyze the impact of different barrier functions on algorithm performance, Figure 8 shows the overall average controllability ratio (CR) and average power loss (PL) for the six MADRL algorithms under the influence of different barrier functions. It can be seen from the figure that within the first 180 iterations, when function $f_{2}\;$ is used as the barrier function, the improvement speed and stability of CR are significantly better than when function $f_{1}$ is used; after the number of iterations exceeds 180, the performance of the two in terms of the CR metric gradually converges. This phenomenon indicates that $f_{2}$ can provide a larger gradient in the voltage violation region, enabling the agents to correct voltage deviations faster and approach the rated value in the early training stages, thereby accelerating the improvement of system controllability.

Regarding the PL metric, algorithms using function $f_{2}$ perform better than those using $f_{1}$. The reason is that $f_{2}$ provides a smaller gradient response when the voltage is close to the rated value, preventing excessive reactive power adjustment during the steady-state phase, thus reducing energy waste and improving overall energy efficiency.

**Figure 9:**
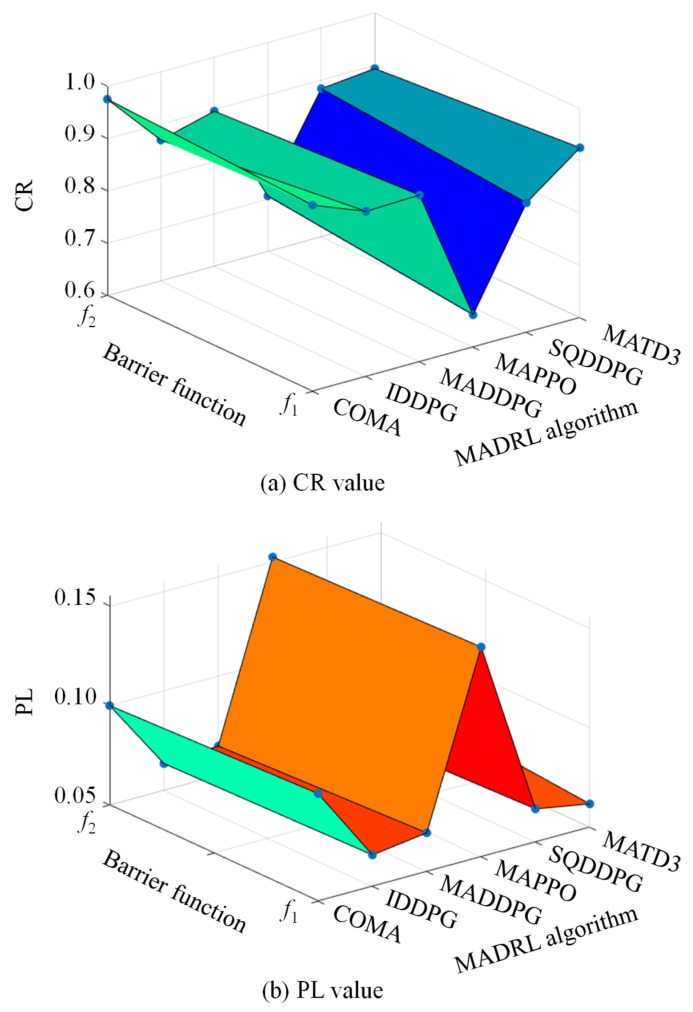
CR and PL under different MADRL algorithms and obstacle functions.

To deeply analyze the relationship between MADRL algorithm performance and barrier function configuration, training was conducted under different combinations of algorithms and barrier functions, and the corresponding CR and PL were statistically analyzed after the 400th iteration. The specific performance metrics for each combination are summarized in Table 1, showing the numerical comparison for CR and PL, respectively. Furthermore, to visually present the combined impact of algorithm type and barrier function on system performance, 3D surface plots were drawn, as shown in Figure 9. Here, the *X*-axis represents the six algorithm types (including COMA, IDDPG, MADDPG, MAPPO, SQDDPG, and MATD3), the *Y*-axis represents the selected barrier function type, and the *Z*-axis corresponds to the values of CR and PL, respectively. In particular, CR reflects the algorithm’s capability to maintain all node voltages within the admissible range under stochastic load–PV fluctuations, whereas PL indicates the additional reactive-power-induced system losses incurred during such control operations. A higher CR signifies stronger voltage feasibility and robustness, while a lower PL implies more economical reactive dispatch.

Combining the results from Table 1 and Figure 9, it can be seen that when using the same type of MADRL algorithm, different barrier functions have a significant impact on CR. Specifically, when the barrier function is $f_{1}$, algorithms such as IDDPG, MADDPG, and MATD3 perform better in the CR metric, achieving higher voltage controllability with fewer iterations; when the barrier function is $f_{2}$, algorithms such as COMA, MAPPO, and SQDDPG show more prominent CR metrics, indicating their stronger adaptability to voltage constraints under the influence of this type of barrier function. These results indicate that the design of the barrier function has an important impact on control performance, and different algorithm structures and learning mechanisms respond differently to the gradient characteristics of the barrier function, leading to differences in system controllability performance.

On the other hand, under the same algorithm type, different barrier functions also have a noticeable impact on PL. When the barrier function is $f_{2}$, algorithms such as COMA, IDDPG, MADDPG, MAPPO, and SQDDPG all achieve lower PL, indicating that this function helps to reduce the system’s reactive power adjustment range and improve energy efficiency; when $f_{1}$ is used as the barrier function, only the MATD3 algorithm shows good PL optimization results. This result further indicates that the choice of barrier function not only affects voltage control accuracy but also directly affects the system’s energy utilization efficiency. Therefore, the form of the barrier function should be reasonably selected considering the learning characteristics of the algorithm and the operational characteristics of the system to achieve the optimal balance between voltage controllability and power loss.

### 4.5. Comparison with Traditional Control Methods

**Figure 10:**
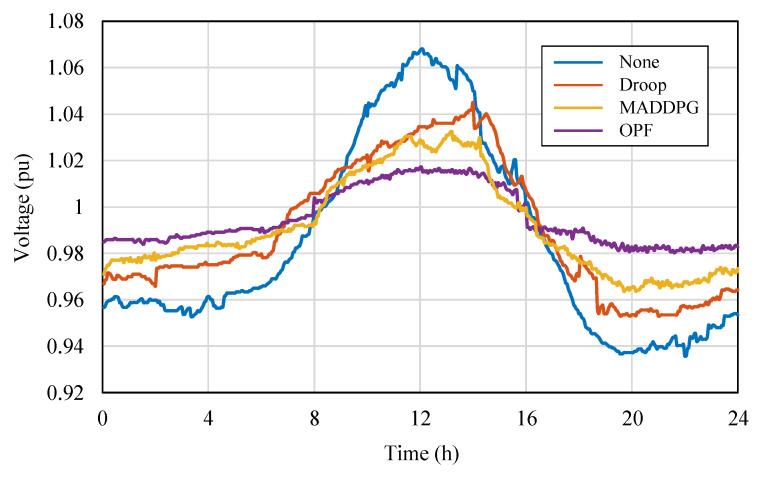
Bus voltage curves under different control methods.

**Figure 11:**
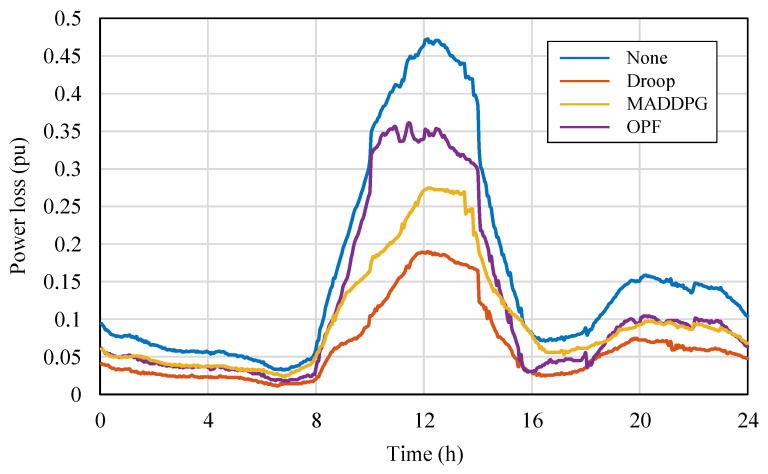
Power Loss Curves Under Different Control Methods.

To compare the differences in control performance between MARL algorithms and traditional control methods in PV distribution networks, targeted tests were conducted based on the modified IEEE 33-node distribution network. Considering simplified experimental configuration and highlighting representativeness, MADDPG, which performed excellently in both CR and PL metrics, was selected as the representative of the MARL method; traditional control methods selected two typical strategies for comparison: Optimal Power Flow (OPF) control and droop control. The tests mainly focused on the bus voltage fluctuations and power loss performance during a one-day operation period with PV integration into the grid. The relevant results are plotted in Figure 10 and Figure 11, respectively. Figure 10 shows the changes in bus voltage over time under the action of different control methods, used to evaluate their control capability for voltage stability; Figure 11 shows the corresponding trend of the overall system power loss during the period, used to quantify the differences in energy utilization efficiency among the methods.

From Figure 10, it can be seen that without control, the system voltage exceeds the safe operating range during both the midday high irradiation and nighttime low load periods, indicating obvious voltage instability risks in the distribution network with high PV penetration. After introducing control, all three methods (MADDPG, OPF, and droop control) can maintain the voltage within the safe range, but their control characteristics differ significantly. The OPF method performs the most steadily in terms of voltage stability, with the smallest bus voltage fluctuation amplitude; the MADDPG method follows, achieving strong dynamic tracking capability while ensuring voltage safety; the voltage regulation effect of droop control is relatively poor, with voltage deviations occurring in some periods, reflecting its delayed response in dealing with rapid power fluctuations.

From Figure 11, it can be further observed that the droop control method has the highest power loss, MADDPG’s power loss is between the other two, and OPF has the lowest power loss. This result reflects the essential differences in the control mechanisms of the three methods: droop control, as a distributed local strategy, has the advantages of fast response and simple structure but lacks global coordination capability, making it difficult to achieve optimal energy efficiency; MADDPG, relying on a multi-agent distributed learning mechanism, achieves global coordination based on local information interaction, combining dynamic responsiveness and adaptability, achieving a good balance between voltage stability and energy efficiency; OPF belongs to the centralized optimization method, relying on a complete system model and global information, and can theoretically obtain the global optimal solution, thus performing best in terms of voltage stability and power loss. The droop-controlled system exhibits noticeably higher power losses, with peak values approaching 0.35 pu during the midday high-PV interval. This behavior arises from the inherent characteristics of droop regulation under weak-grid conditions. Because droop control responds solely to local voltage measurements, each inverter adjusts its reactive power independently without global coordination. When PV generation is high and the power flow direction becomes reversed, the uncoordinated reactive-power injections from multiple inverters tend to reinforce each other, creating reactive circulation across adjacent feeders. These interactions significantly increase the current magnitude along several branches, especially in parts of the network with relatively high R/X ratios. The weak-grid environment further amplifies this effect, as limited short-circuit strength makes the system more sensitive to reactive-power oscillations and voltage rise. Consequently, both branch currents and I^2^R losses increase substantially, leading to the observed higher power loss levels in the droop-controlled case.

An additional severe-case scenario is designed to evaluate the necessity and effectiveness of advanced coordinated control strategies under weak-grid conditions. In this case, the PV and load profiles are amplified to produce stronger midday generation peaks and deeper evening troughs, resulting in larger active and reactive power swings across the distribution network. Such conditions are representative of real-world weak-grid environments, where limited upstream voltage support and rapid PV fluctuations can push the system close to operational limits even when the base case appears acceptable.

**Figure 12:**
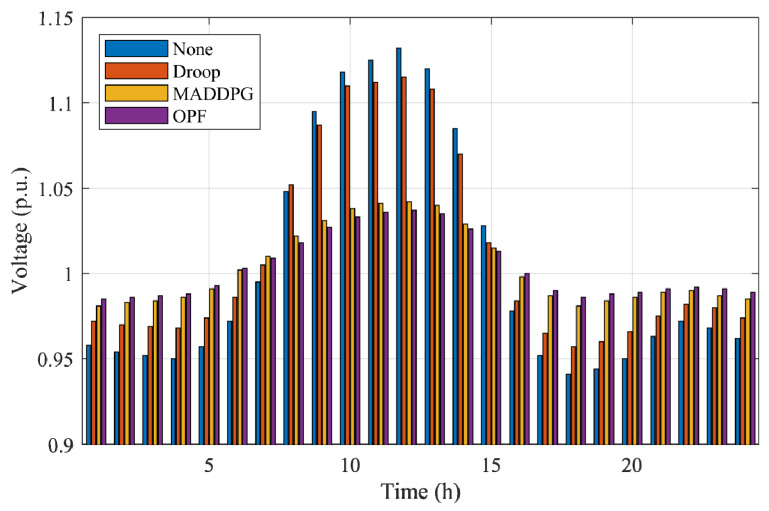
Bus voltage profiles under the four control methods in the severe-case scenario.

**Figure 13:**
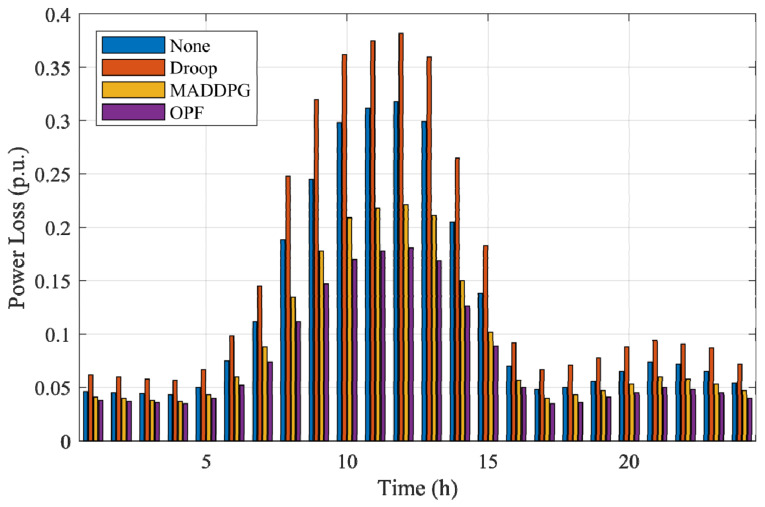
Power loss under the four control methods in the severe-case scenario.

Figure 12 illustrates the 24 h bus voltage trajectories under the four control strategies (None, Droop, MADDPG, and OPF) in this severe case. The figure shows the instantaneous voltage levels of the most sensitive bus, enabling a direct comparison of each method’s capability to suppress over-voltage during high-irradiance periods and maintain stability during low-load conditions. Figure 13 presents the corresponding 24 h total power loss curves, reflecting the energy-efficiency implications of different control actions across the same operating horizon.

From Figure 12, the uncontrolled system exhibits pronounced voltage rise, with peak values exceeding 1.12 p.u. during the midday PV peak. Droop control reduces the magnitude of the over-voltage but still exceeds 1.10 p.u. in multiple intervals due to its purely local and uncoordinated reactive-power behavior. In contrast, both MADDPG and OPF maintain all bus voltages strictly within the 0.95–1.05 p.u. safety band throughout the entire day. MADDPG demonstrates fast dynamic adaptation to rapidly changing PV injections, while OPF provides the most stable trajectory owing to its centralized global optimization. Figure 13 further shows that the severe-case operating conditions lead to significantly higher power losses for the uncontrolled and droop-controlled systems, with values rising above 0.30 p.u. during the midday peak. These losses originate from voltage deviations and reactive-power circulation under reverse-flow periods. MADDPG substantially reduces power losses by learning coordinated reactive-power responses among agents, approaching the performance of OPF, which achieves the lowest loss due to its explicit optimization of system-wide objectives.

To more intuitively illustrate the differences between the proposed MADRL method and traditional control strategies, Table 2 provides a detailed comparison across dimensions such as model dependency, communication requirements, computational mode, and control performance.

Table 3 shows the computation time per control step for different methods. In terms of computational efficiency, the three control methods exhibit fundamentally different behaviors. Droop control achieves the fastest execution, requiring only simple algebraic operations based on local voltage measurements, which results in an average computation time of approximately 0.15 ms per step. The proposed MADDPG method also maintains a low online computational burden, as decentralized execution is implemented through a lightweight neural-network forward pass, yielding an average computation time of 3.8 ms per step. In contrast, OPF entails solving a constrained nonlinear optimization problem at every control step using the full network model, which leads to a substantially higher computation time of around 1.25 s. This comparison demonstrates that MADDPG offers a favorable balance between computational efficiency and control performance, achieving fast online execution while providing significantly improved voltage regulation capability.

### 4.6. Discussion of Numerical Results and Implications

Based on the case studies presented above, this section provides an in-depth discussion of the numerical results and their implications for voltage control in weak grids. First, the numerical results indicate that algorithm selection is critical for control effectiveness. According to the statistical data in Table 1, the MADDPG algorithm combined with barrier function *f*_1_ achieves the highest Controllability Ratio (CR = 91.9%) and low Power Loss (PL = 0.0695 p.u.). In contrast, under the same test conditions, the poorest performing algorithm, MAPPO, yields a CR of only 66.2% and a significantly higher PL of 0.1554 p.u. This implies that MADDPG reduces the risk of voltage violation by approximately 25% and cuts network losses by more than 50% compared to MAPPO. This significant difference confirms that under the decentralized execution architecture, algorithms utilizing a Critic network for centralized value evaluation (such as MADDPG) cope more effectively with the strong coupling characteristics inherent in distribution network voltage control than simple policy gradient methods (like MAPPO), thereby maintaining higher control accuracy in complex weak grid environments.

Secondly, the design of the barrier function plays a significant role in regulating system energy efficiency. Comparing the experimental results of the two barrier functions reveals that the quadratic barrier function *f*_2_ significantly enhances energy efficiency. Statistical data show that Power Loss (PL) decreases for most algorithms when using *f*_2_; for instance, when MADDPG is combined with *f*_2_, PL further drops to 0.0648 p.u. The physical interpretation is that *f*_2_ provides a gentler gradient within the safe voltage region (0.95–1.05 p.u.), allowing agents to “learn” to avoid unnecessary reactive power regulation when the voltage is within the safe range, thereby preventing the waste of inverter capacity and an increase in line losses. This finding highlights the importance of introducing adaptive gradient mechanisms in reward function design, offering valuable guidance for distribution system operators aiming for economic operation.

Finally, through comparison with traditional methods, MADDPG exhibits immense potential for practical application. While Optimal Power Flow (OPF) achieves the lowest losses, it suffers from long computation times and heavy reliance on accurate physical models; conversely, droop control exhibits obvious steady-state voltage deviations. MADDPG maintains millisecond-level online response speeds while approximating the control effectiveness of OPF (with voltage fluctuation curves highly coinciding), without relying on global real-time communication. This suggests that the proposed method is feasible for replacing traditional local control in practical engineering. Particularly in high-PV-penetration scenarios, it can resolve voltage violations and improve overall grid economy with extremely low online computational costs, achieving an optimal balance between voltage stability and energy efficiency.

## 5. Conclusions

This study aims to enhance voltage stability in weakly supported distribution networks by developing a data-driven multi-agent deep reinforcement learning (MADRL) approach for coordinated control of photovoltaic (PV) clusters. The voltage regulation problem is formulated as a Dec-POMDP, and six representative MADRL algorithms are evaluated on a modified IEEE 33-bus system. Barrier-function-based reward shaping and comparisons with traditional voltage control methods are incorporated to comprehensively assess performance. The main conclusions are as follows:

(1) Effectiveness of the MADRL framework. All six MADRL algorithms achieve stable convergence, demonstrating that agents can autonomously learn effective voltage regulation strategies. Among them, MADDPG exhibits the best overall performance, achieving a Controllability Ratio of 91.9% and a Power Loss of 0.0695 p.u. These results indicate that the learned policy can reliably maintain voltage safety while reducing operational losses, which is especially critical under weak-grid conditions.
(2) Impact of barrier-function design. The proposed quadratic barrier function effectively accelerates voltage recovery in violation regions while avoiding unnecessary reactive power adjustment within the safe voltage band. This contributes to a more economical and smoother control process, emphasizing the importance of reward shaping in practical grid control applications.
(3) Comparison with traditional control methods. The MADDPG-based strategy provides a favorable balance between voltage stability and energy efficiency, outperforming droop control and approaching the optimal behavior of OPF while retaining millisecond-level online inference. This illustrates the feasibility and advantages of data-driven coordinated control for real-world distribution networks with high PV penetration.

Despite its effectiveness, the proposed method still has several limitations. The decentralized execution assumes instantaneous and noise-free local measurements, which may not fully reflect realistic sensor disturbances. In addition, the method is tested under a fixed network topology within each control cycle, and its robustness against abrupt topological changes or communication interruptions has not yet been evaluated. Future work will focus on extending the algorithm to time-varying and uncertain topologies, incorporating measurement noise models, and exploring online adaptation mechanisms to further improve resilience and practical applicability in real distribution grids.

## Figures and Tables

**Table 1:** CR and PL under different types of MADRL algorithms and different obstacle functions.

Index	Function	COMA	IDDPG	MADDPG	MAPPO	SQDDPG	MATD3
CR	*f*_1_	0.956	0.916	0.919	0.662	0.848	0.925
	*f*_2_	0.975	0.870	0.896	0.706	0.883	0.893
PL	*f*_1_	0.1040	0.0658	0.0695	0.1554	0.0667	0.0618
	*f*_2_	0.0999	0.0634	0.0648	0.1524	0.0630	0.0673

**Table 2:** Characteristic Comparison between Proposed MADRL and Existing Control Methods.

Feature Dimension	Droop Control	Centralized OPF	MADRL(MADDPG)
Model Dependency	Model-free (Based on local measurements)	High (Requires accurate topology and parameters)	Model-free (Learns via environment interaction)
Communication	None/Low (No real-time comms)	High (Global real-time data acquisition)	Low (Global for training, Local only for execution)
Decision Mode	Decentralized	Centralized	Centralized Training, Decentralized Execution (CTDE)
Online Complexity	Very Low (Simple algebraic calculation)	High (Iterative solving of non-convex optimization)	Low (NN forward inference, milliseconds)
Global Optimality	Weak (Local regulation only)	Strong (Theoretical global optimum)	Strong (Approximates global optimum, balances voltage and loss)
Main Limitation	Large steady-state error, lack of coordination	Vulnerable to single-point failure, high latency	Long training time (offline)

**Table 3:** Computation Time per Control Step for Different Methods.

Method	Droop Control	Centralized OPF	MADRL (MADDPG)
Computation Time per Step	0.15 ms	3.8 ms	1.25 s

## Data Availability

The data presented in this study are available on request from the corresponding author.
